# Neurobiology of Memory and Anxiety: From Genes to Behavior

**DOI:** 10.1155/2007/78171

**Published:** 2007-01-10

**Authors:** Allan V. Kalueff

**Affiliations:** Laboratory of Clinical Science, Division of Intramural Research Program (DIRP), National Institute of Mental Health (NIMH), Bethesda, MD 20892-1264, USA

## Abstract

Interaction of anxiety and memory represents an essential feature of CNS functioning. This paper reviews experimental data coming from neurogenetics, neurochemistry, and behavioral pharmacology (as well as parallel clinical findings) reflecting different mechanisms of memory-anxiety interplay, including brain neurochemistry, circuitry, pharmacology, neuroplasticity, genes, and gene-environment interactions. It emphasizes the complexity and nonlinearity of such interplay, illustrated by a survey of anxiety and learning/memory phenotypes in various genetically modified mouse models that exhibit either synergistic or reciprocal effects of the mutation on anxiety levels and memory performance. The paper also assesses the putative role of different neurotransmitter systems and neuropeptides in the regulation of memory processes and anxiety, and discusses the role of neural plasticity in these mechanisms.

## 1. INTRODUCTION

Pathologic anxiety is a complex stress-related disorder which includes generalized anxiety, panic, social anxiety, agoraphobia, posttraumatic stress, and obsessive-compulsive disorders [1–5]. There are many animal (experimental) paradigms that model different subtypes of human anxiety [6–10]. In addition to anxiety, stress has long been known to affect animal and human cognitions [11–14], raising the possibility that memory and anxiety interact.

Numerous studies have outlined behavioral, physiological, pharmacological, and genetic aspects of memory-anxiety interaction [13, 15–20]. Since memory consolidation and anxiety both require brain arousal, it has been considered as promnestic and anxiogenic, whereas brain inhibition is amnestic and anxiolytic; review [12, 21, 22]. However, classic works of Yerkes and Dodson [14], as well as many subsequent studies [23–30], have shown that memory and stress interplay in a more complex, type-specific, and nonlinear manner. Here we will analyze the available clinical and experimental data in order to examine (with a particular focus on neurogenetics) the links between anxiety and memory functions.

Transgenic and mutant animals, including tissue-specific and inducible knockout mice, represent a valuable tool for biomedical brain research [31–34] powered by extensive on-line databases [8, 9]. Table 1 summarizes anxiety and memory/learning phenotypes in various genetically modified mouse models, including mutant mice lacking or over-expressing receptors of various neuromediators, neuropeptides, and some brain proteins mediating neuroplasticity. Several important conclusions can be made based on these findings. A common situation when the same mutation leads to altered anxiety and memory phenotypes (Table 1) confirms overlapping of the two domains at genetic (in addition to behavioral and pharmacological [12, 13]) levels. While many mutants show synergetic alterations of memory and anxiety, there are also data on reciprocal effects of some mutations (Table 1), confirming a complex nonlinear nature of memory-anxiety interplay. Moreover, as can be seen in this table, different subtypes of memory seem to be differentially influenced by altered anxiety, further contributing to the complexity of the problem discussed here. While this paper will not offer a simple solution for complex animal or human phenotypes, its aim is to discuss how different brain systems may interact in determining anxiety and memory phenotypes.

## 2. NEUROCHEMISTRY AND NEUROGENETICS OF MEMORY AND ANXIETY

Cholinergic synaptic transmission has long been implicated in learning, memory, and anxiety [36, 92]. Neuronal nicotinic (N) acetylcholine (ACh) receptors are hetero-oligomers (formed by five of 11 known *α* and *β* subunits) mediating anxiolytic-like effect of nicotine [35]. Their loss has also been noted for Altzheimer's and Parkinson's patients with impaired cognitive functions [35], collectively implicating these receptors in both memory and anxiety. In line with this, increased anxiety and impaired memory were reported in mice lacking *α*4 subunit of N-type Ach receptor (Table 1). Mice lacking the receptor's *β*2 subunits (predominant in hippocampus) showed impaired avoidance learning, but normal spatial learning in Morris water maze [37]. Surprisingly, ablation of *α*7 subunits (also rich in hippocampus) leads to no or very mild alterations in anxiety (open field test) and memory (unaltered acoustic startle habituation and Pavlovian conditioning, but faster finding a platform in the Morris water maze) [36]. Taken together, this suggests that various subtypes of ACh receptors may play different roles in memory-anxiety interplay. Notably, RS-1259, a newly synthesized inhibitor of acetylcholinesterase [93], elevated ACh levels in hippocampus and improved memory in mice, suggesting that targeting brain ACh may lead to effective therapy of neurodegenerative disorders. The same drug also inhibited serotonin transport [93], implying that altered serotonergic system may also contribute to these effects (see further).

Gamma-amino butyric acid (GABA) is the primary mediator of inhibitory neurotransmission, acting through ionotropic A and metabotropic B type receptors. GABA-A receptors are Cl-channels composed of five subunits (from eight families: *α*1-*α*6, *β*1-*β*3, *γ*1-*γ*3, *δ*, *ɛ*, *π*, *θ*, and *ρ*1-*ρ*3) with multiple binding sites for positive (GABA agonists, barbiturates, benzodiazepines, steroids, and ethanol) and negative (GABA-A antagonists, neurosteroid antagonists, benzodiazepine inverse agonists, and chloride channel blockers) modulators [4, 12, 94–97]. GABA has long been implicated in anxiety [80, 97–101]. In both humans and animals, positive modulators of GABA receptors generally possess anxiolytic activity while negative modulators produce anxiogenic-like effects. Moreover, various GABA analogs and agents affecting transmitter metabolism to enhance GABAergic tone have been reported to exert anxiolytic effects [98, 102–107]. The role of GABA in learning and memory has also been widely recognized [28–30, 90, 100, 108–112]. Three comprehensive reviews particularly [12, 17, 113] emphasize the role of central GABA in memory-anxiety interplay, noting amnestic/anxiolytic effects of positive, and opposite profiles of negative, GABA modulators (also see [27–30, 111, 114, 115] for details).

Mounting neurogenetic data further implicates GABA in memory and anxiety. GABAergic genes are associated with anxiety (*α*2, *α*3, *α*4, *α*6, *β*1, *γ*1, and *γ*2) [95, 96, 116, 117] and memory (*α*5) [48, 49, 118]; see Table 1. Downregulation of *α*1, *α*4, *α*5, *α*6, *γ*1, *δ* genes was reported in anxious versus nonanxious rat strains [119]. Other studies show reduced expression of rat *α*2, *γ*1, or *δ* subunits after fear conditioning [79] and chronic unpredictable stress [120]. In humans, treatment-resistant depression with anxiety was linked to a mutant *β*1 subunit gene [121], whereas positive genetic associations were found between GABA-A subunits genes and neuroticism (*α*6 [122]), posttraumatic stress disorder with anxiety and depression (*β*3 [123]), and hormonal/autonomic stress responses (*α*6 [124]).

Recent clinical and experimental data outline the role of GABA and GABA-ergic genes in amygdala and hippocampus (Table 2); the brain areas involved in the regulation of both memory and anxiety [125, 126]. In addition to receptors, these domains are also influenced by GABA metabolism. While specific amygdalar reduction in expression of GABA-synthesizing enzyme was observed in animals during learning [126], spatial learning was impaired in rats following anxiolytic GABA transporter inhibitor tiagabine [127]. Collectively, these findings confirm that central GABA is a key mediator regulating anxiety and memory, and that GABAergic genes, metabolism, and/or subunit-specific GABAergic drugs [100, 128–132] may modulate such interplay.

Glutamate receptors mediate most excitatory CNS neurotransmission. There are several known subtypes of metabotropic glutamate receptors which are coupled to G-proteins and exert their effects via second messenger signaling pathways. Genetic ablation of glutamate subtype 7 receptors in mice impairs their performance in two distinct amygdala-dependent paradigms [54] and inhibits hippocampal neurotransmission [133], suggesting that both structures are involved in glutamate-mediated mechanisms of memory and anxiety. Consistent with this, glutamate receptor densities positively correlate with spatial learning abilities in mice [90].

Several recent clinical and experimental data also show that central dopaminergic system plays a role in the regulation of memory and anxiety, including fear conditioning [134, 135]. In line with this, a recent quantitative trait loci study showed that cognitive functions (intertrial habituation) of 25 inbred mouse strains were linked to a region on chromosome 15 mapping dopamine D1 and D2 receptors [136].

Serotonin and its receptors have long been implicated in memory and anxiety in both humans [38, 122, 134, 137, 138] and animals [1, 139–144]. In addition to receptors (Table 1), other factors include serotonin homeostasis and metabolism. Serotonin is removed from the synaptic cleft by a specific membrane transporter protein (SERT [31, 145]), representing an important target for various manipulations. For example, pharmacological inhibition of SERT leads to elevated hippocampal serotonin levels and improved memory [93]. While genetic ablation of SERT in mice is widely used as a model of anxiety [47, 145–148], these mice display increased poststress responsivity [149], indirectly implying a better memory for aversive stimuli. Clearly, further studies are needed to assess the link between SERT and cognitive abilities in animals, and its relevance to human brain dysfunctions. Overall, human anxiety-related traits seem to generally facilitate cognitive functions (e.g., acquisition of conditioned fear), and such interplay is partially serotonergically mediated [134].

Strengthening this notion, genetic variations in SERT have been linked to strain differences in emotional learning in rats [150]. In humans, SERT has also been implicated in anxiety and cognitions. For example, SERT polymorphisms have been associated with anxiety-related personality traits [122, 151], amygdalar reactivity [152–154], cognitive abilities [36, 155], and altered hippocampal neurochemistry [137]. In line with this, Caspi et al. [156] recently established that human SERT polymorphisms modulate the effect of life stress on stress-related CNS pathogenesis, while Fox et al. [157] found association of SERT polymorphisms with children behavioral inhibition—a temperamental construct predicting anxiety.

Importantly, brain catecholamines do not act individually in the brain, interact at different levels with each other, and with other brain molecules [147, 148]. Antipanic drug phenelzine (a nonselective inhibitor of monoamine oxidase MAO A/B which elevates brain norephinephrine, dopamine, and serotonin levels) also exerts mnemotropic effects [19]. MAO A/B knockout mice (demonstrating phenotype similar to the effect of phenelzine) display robust anxiety phenotype but unaltered working memory (Table 1), as assessed by their open field habituation [57]. In contrast, MAO B inactivation in mice leads to increased anxiety, unaltered spatial working memory in Y-maze, but reduced habituation to the forced swim test 4 weeks after the initial trial [56]. Collectively, these data confirm the notion that anxiety and memory phenotypes are heterogeneous and may be determined by interactions of various mediator systems. For example, Birzniece et al. [114] recently analyzed the interplay between GABA-active steroids and serotonin in modulating cognitive functions, and Sibille et al. [45] found reduced GABAergic tone in anxious serotonin 5HT-1A receptor knockout mice, also displaying memory deficits [44].

## 3. NEUROPEPTIDES AND NEURAL PLASTICITY ISSUES

In addition to mediators, brain neuropeptides play a key role in modulation of memory and anxiety. For example, mutants lacking receptors of “anxiogenic” cotricotropin releasing factor (CRF) display a predictable reduction of anxiety accompanied by reduced cognitive performance during the retrieval trial in the Y-maze (Table 1). Overall, these findings are in line with numerous data implicating CRF in both anxiety and memory, and suggest that novel antistress mnemotropic drugs may be created based on targeting central CRH system [58, 167]. In contrast, mutant mice with reduced sensitivity of thyroid receptors [60] display increased anxiety but reduced memory (Table 1), demonstrating that not always various manipulations exert synergetic effects on these two processes. Interestingly, while CRF has been traditionally linked to memory and anxiety, nonanxiogenic doses of CRF type 1 and 2 receptor agonist urocortin produced anxiety (accompanied by amygdalar hyperexcitability) after 5 daily intra-amygdalar infusions in rats [168]. These results indicate that CRF-induced synaptic plasticity, in addition to anxiety and memory processes, may be involved in pathogenesis of emotional disorders (also see [169] for review).

Pituitary adenylate cyclase-activating polypeptide (PACAP) is another important regulator of synaptic plasticity, neurotrophins, neuromediators, and neuronal differentiation [67, 68]. It binds to a highly selective type 1 receptor (PAC1), widely distributed in the limbic system, including amygdala and hippocampus. Since mice lacking PAC1 demonstrate reduced anxiety and impaired memory (Table 1), PACAP/PAC1 system may be directly involved in the regulation of memory-anxiety interplay. Clearly, further studies are needed to explore this interesting aspect in detail, including its relation to PACAP/PAC1-mediated neuroimmuno-modulation and neuroprotection [170] and impairment in mossy fiber long-term potentiation [68].

Glial Ca-binding protein S100B also plays an important modulatory role in memory. S100B knockout mice display strengthened synaptic plasticity, enhanced long-term potentiation, and spatial memory in Morris water maze, while mice over-expressing this protein exhibit the opposite phenotype [62]. Importantly, these findings show that both neurons and glial cells modulate brain synaptic plasticity, and that glial-neuronal interactions must also be considered in examining memory-anxiety interplay in the CNS.

Protein kinase C (PKC) *γ* is an enzyme highly expressed in the limbic system—the brain structure that regulates both memory and anxiety [63, 64]. Since PKC*γ* plays an important role in neural plasticity, modulation of neurotransmitter release, and neuronal excitability, its genetic ablation in mice predictably affects their anxiety and learning (Table 1). Mechanisms underlying these effects are still unknown but most likely include postsynaptic modulation of central GABA-A and serotonergic 5HT2 receptors [64].

From various brain proteins essential for synaptic vesicle trafficking, ras-associated binding proteins, such as Rab3a [70, 171], deserve special attention in relation to memory and anxiety. Using Rab3a knockout (−/−) and Ebd (loss-of-function) Rab3a mutant mice, a recent study has shown that Rab3a −/− mice display reduced cued fear conditioning, while Ebd mutants show both reduced anxiety and cued fear conditioning (Table 1), accompanied by altered hippocampal and cortical expression of Rab3a [69]. D'Adamo et al. [70] reported that Rab3a −/− mice display deficits in short- and long-term synaptic plasticity in the mossy fiber pathway, normal acquisition but several mild impairments in other memory tasks (Table 1), accompanied by increased locomotion and reduced anxiety. Collectively, these data implicate protein modulators of synaptic transmission (such as Rab3a) in the regulation of memory and anxiety, also enabling further dissection of molecular domains involved in their regulation.

Another recent study demonstrated that Rab3a is required for brain-derived neurotrophic factor (BDNF)-induced synaptic plasticity [172], implying functional interplay between the two molecules involved in brain plasticity. Indeed, BDNF is a key neurotrophic factor, acting through trkB receptor to regulate brain growth, differentiation, and functioning [32, 160, 173]. While an early study showed no anxiety or memory effects of BDNF genetic ablation in mice, numerous other data did reveal such actions (see Table 3 for details), also implying BDNF role in aversive memories [158, 162]. Consistent with this, spatial learning induces BDNF and trkB expression in activated brain areas, while BDNF inactivation markedly impairs spatial learning [32, 165]. In addition, mutant mice with reduced BDNF levels display impaired learning and memory in some tasks [159], whereas increased mouse BDNF signaling by trkB overexpression improves memory [165].

BDNF is rich in hippocampus and amygdala, and its administration improves rat short-term spatial memory and reduces anxiety [163]. In contrast, the same study revealed increased anxiety on trial 2 in BDNF-treated rats, suggesting that different types of anxiety may differently interplay with BDNF-modulated memories. In line with this, increased BDNF signaling in mice over-expressing trkB produced anxiolysis [165], while stress and anxiety correlate with memory deficits and reduction in brain BDNF [174, 175]. Moreover, Rattiner et al. [176, 177] have recently outlined the crucial role of BDNF and its receptors in hippocampal and amygdala-dependent learning (including fear conditioning—another potential mechanism underlying BDNF modulation of memory and anxiety).

Overall, human data strikingly parallel animal data on BDNF role in memory and anxiety (Table 3). For example, functional BDNF polymorphisms have been associated with anxiety-related personality traits [178], hippocampal volume in healthy volunteers [179], and episodic memory [180]. Taken together, these data confirm the important role of BDNF in memory, anxiety, and their interplay. Given the important role of BDNF in brain plasticity [173], behavior-modulating properties of this molecule seem to be particularly intriguing.

Importantly, brain mediators seem to cooperate with BDNF in modulating brain functions. For example, BDNF interacts with cholinergic, dopaminergic, serotonergic systems, and SERT [181–184] whose involvement in memory and anxiety has already been discussed. Analyses of human quantitative trait loci associated with cognitive functions also pointed to genes encoding BDNF, ACh, and glutamate receptors [185]. From this point of view, it is interesting that heterozygous BDNF knockout mice display unaltered or little anxiety and rather mild alterations in memory (Table 3), accompanied by altered hippocampal ACh but unaltered catecholamine levels [160]. In contrast, simultaneous ablation of BDNF and SERT alleles exacerbates anxiety in double knockout mice and reduces hippocampal serotonin levels [147, 186], confirming an important functional interplay between BDNF and serotonin in the brain [181]. Extending original findings of Caspi et al. [156], a recent study has examined BDNF/SERT genes' interactions in depressed children, reporting that a combination of met-BDNF allele with two short SERT alleles was associated with higher depression in maltreated children [187]. Notably, this situation strikingly resembles experiments of Ren-Patterson et al. [186] in mice, indirectly supporting the notion that depression as well as specific anxiety-related traits (i.e., social anxiety or posttraumatic stress) may also be involved in BDNF-SERT interplay; also see [158, 162] for discussion.

## 4. CONCLUSIONS

**Figure 1:**
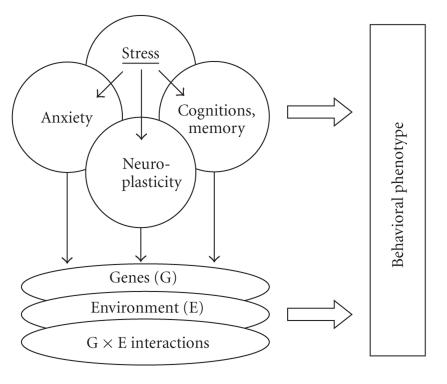
Stress, memory, and anxiety interplay.

As already mentioned, memory and anxiety do not always follow synergetic “high anxiety-better memory” rule, indicating that more complex nonlinear relations exist between these behavioral domains. Moreover, not always altered anxiety is seen together with altered memory, as vise versa (Table 1), suggesting that under certain circumstances both domains may be affected independently. Likewise, memory (as well as anxiety) must not be considered as a single entity, and clearly represents a complex multidimensional domain. However, it is important to understand that memory and anxiety represent two overlapping CNS processes that closely interact at different levels, including brain neurochemistry, circuitry, pharmacology, and various genes, as discussed here in detail. For such interactions, clinical findings strikingly parallel animal experimentation data, showing how these factors (in addition to environmental influences) may affect memory and anxiety. Both neuronal and glial cells, as well as brain mediators, neuropeptides, and other key proteins, cooperate in the regulation of memory and anxiety (Figure 1). Finally, brain plasticity factors (Figure 1) appear to play an important role in fine-tuning of memory-anxiety interplay, collectively contributing to the complexity of behavioral phenotypes.

## Figures and Tables

**Table 1:** Mouse mutagenesis data on memory and anxiety phenotypes [8]; see text for details. KO: knockout (−/−), HZ: heterozygous (+/−) mice. (↑: increased, ↓: reduced, 0: no effects, ↔: mixed or unclear results. CRF: corticotropin-releasing factor, MAO: monoamine oxidase A/B, FXR1: fragile X-related protein 1, PACAP: pituitary adenylate cyclase activating polypeptide, Rab3a: *ras*-associated binding 3a protein.)

Mouse models		Effects on		References
		Anxiety	Memory/learning

Neurotransmitters Acetylcholine	N-receptor *α*4 subunit KO mice	↑	↓ within-trial habituation	[35]
	N-receptor *α*7 subunit KO mice	0 (↓)	0 fear conditioning, spatial learning	[36]
	N-receptor *β*2 subunit KO mice	—	↓ avoidance learning, 0 spatial learning	[37]
	
Serotonin	5HT-1B receptor KO mice	↓	↑ long-term and short-term memory, 0 habituation	[38–42]
	5HT-1A receptor KO mice	↑	↓ hippocampal-dependent learning, 0 habituation	[40, 43–45]
	5HT-5A receptor KO mice	↓	0 inter- and within-trial habituations	[46]
	Serotonin transporter KO mice	↑	↔ within-trial habituation	[47]
	
GABA (also see Table 2)	GABA-A *α*5 subunit KO mice	0	↑ hippocampal-dependent trace conditioning, 0 delayed or contextual conditioning	[48]
	GABA-A *γ*2 subunit HZ mice	↑	↑ cued fear conditioning, 0 spatial memory	[49]
	
Histamine	Histamine H3 receptor KO mice	↓	0 habituation, ↑ spatial memory and learning, higher resistance to amnestic effects of scopolamine	[50, 51]
	
Glycine	Glycine transporter 1 brain-selective disruption	0	↑ aversive Pavlovian conditioning	[52]
	
Glutamate	B subunit ionotropic receptor KO mice		↓ olfactory memory (rescued by selective expression in hippocampus)	[53]
	Metabotropic subtype 7 receptor KO mice	↓	↓ cued fear response and conditioned taste aversion	[54]
	A type receptor KO mice	↑	↓ spatial working memory (alternation)	[55]
	
Related models	MAO B targeted inactivation	↑	0 working memory, ↓ long-term memory	[56]
	MAO A/B KO mice	↑	0 within-trial habituation	[57]
	
Neuropeptides and other brain proteins	CRF receptor 1 KO mice	↓	↓ spatial recognition memory	[58]
	Thyroid hormone *α*1 receptor mutations	↑	↓ olfactory recognition memory, contextual fear conditioning	[59, 60]
	Neuropeptide Y KO mice	↓	↓ attention training test performance	[61]
	Brain-derived neurotrophic factor (mice)	↔	↔	Table 3
	Glial protein S100B KO mice		↑ fear conditioning, spatial memory	[62]
	Protein kinase C*γ* KO mice	↓	↓ spatial and contextual learning	[63, 64]
	FXR1 KO mice	↓	↓ fear conditioning, spatial memory, 0 habituation	[65]
	Modified *β*-amyloid precursor KO mice	↑	↓ spatial learning, habituation	[66]
	PACAP-type 1 receptor KO mice	↓	↓ associative learning	[67, 68]
	Rab3a KO mice	0 ↓	↓ cued fear conditioning 0 acquisition, mild ↓ spatial reversal learning and spatial working memory	[69] [70]
	Rab3a loss-of-function mutant mice	↓	↓ cued fear conditioning	[69]

**Table 2:** Clinical and preclinical data linking common GABAergic brain areas to pathogenesis of anxiety and depression.

Clinical data	Animal data

Amygdala (anxiety, memory)	
Activation in patients with posttraumatic stress disorder [71], during anticipatory anxiety [72], in adults and adolescents viewing fearful faces; also positive correlation of amygdalar activation and social anxiety scores [73–75].	Reduced anxiety and memory in rats following muscimol injection [76–78]. Reduced expression of GABA-A receptor associated protein^(a)^ after fear conditioning in rats [79]. Increased c-fos expression^(b)^ in rats following anxiogenic drugs [10]. Correlation between anxiety phenotype and reduced GABA-A receptor densities, benzodiazepine binding, and *γ*2 subunit mRNA levels in mice and rats [80–82]. Altered amygdalar electric activity during fear conditioning in mice [83]. Reduced extracellular GABA in mice exposed to conditioned fear stimulus [84].

Hippocampus (memory, anxiety)	
Reduced blood flow in anxious volunteers during phobogenic (versus neutral) visual stimulation [85]. Decreased blood flow in right hippocampus in women with posttraumatic stress disorder [86]	Reduced expression of *α*2 GABA-A receptor subunit 6 hours after fear conditioning in rats [79]. Correlation between anxiety and altered benzodiazepine binding in rats [27, 82]. Reduced expression of *α*1 and *α*2 subunits mRNA in punished rats [87]. Altered volume in anxious HAB (versus low-anxiety LAB) rats [88]. Increased c-fos expression in rats following administration of anxiogenic drugs [10]. Reduced hippocampal allopregnanolone levels in anxious high-vocalizing rats [89]. Correlation between mouse spatial learning abilities and GABA-A receptor densities [90]. Disrupted context-specific fear memory in rats following muscimol injection [91].

^(a)^ Modulates channel kinetics and neurotransmission by promoting GABA-A receptor clustering.

^(b)^Genetic marker of neuronal activation.

**Table 3:** Summary of data showing the role of BDNF in memory and anxiety. KO: knockout (−/−), HZ: heterozygous (+/−) mice. (?: unclear effects. ∗: although authors claimed that anxiety was unaltered in this study, it contradicts the original anxiogenic interpretation of the social defeat model (also see [158]).)

Model	Effects on		References
	Anxiety	Memory/learning

BDNF HZ mice	0	↓ learning (but 0 spatial learning and memory, fear conditioning)	[159], but see [160, 161]

Repeated aggression accompanied by increased BDNF expression in mice	↑*	↑ long-term social aversion	[162]

Mesolimbic-specific BDNF knockdown	↑*	↓ long-term social aversion	[162]
BDNF intrahippocampal injection in rats	↓↑	↑ short-term spatial memory	[163]
BDNF injection to the cortex in rats		↑ long-term memory	[164]
BDNF receptor overexpression in mice	↓	↑ spatial memory and learning	[165]

Forebrain-specific BDNF KO mice	0 ↑?	↓ spatial and nonspatial discrimination learning, 0 contextual fear	[166]

Brain conditional BDNF KO mice	↑	—	[33]
